# Risk of attention‐deficit hyperactivity disorder in offspring of mothers with infections during pregnancy

**DOI:** 10.1002/jcv2.12070

**Published:** 2022-03-10

**Authors:** Kjersti M. Walle, Ragna B. Askeland, Kristin Gustavson, Siri Mjaaland, Eivind Ystrom, W. Ian Lipkin, Per Magnus, Camilla Stoltenberg, Ezra Susser, Michaeline Bresnahan, Mady Hornig, Ted Reichborn‐Kjennerud, Helga Ask

**Affiliations:** ^1^ Norwegian Institute of Public Health Oslo Norway; ^2^ Medical Research Council Integrative Epidemiology Unit University of Bristol Bristol UK; ^3^ Department of Psychology University of Oslo Oslo Norway; ^4^ Promenta Research Center University of Oslo Oslo Norway; ^5^ School of Pharmacy University of Oslo Oslo Norway; ^6^ Department of Epidemiology Columbia University Mailman School of Public Health New York USA; ^7^ Center for Infection and Immunity Columbia University Mailman School of Public Health New York USA; ^8^ Departments of Neurology and Pathology Columbia University Vagelos College of Physicians and Surgeons New York USA; ^9^ Centre for Fertility and Health Norwegian Institute of Public Health Oslo Norway; ^10^ Department of Global Public Health and Primary Care University of Bergen Bergen Norway; ^11^ New York State Psychiatric Institute New York USA; ^12^ Department of Medicine University of Oslo Oslo Norway

**Keywords:** ADHD, infections, MBRN, MoBa, pregnancy

## Abstract

**Background:**

Maternal infections during pregnancy are common events that have been suggested to be risk factors for Attention‐deficit hyperactivity disorder (ADHD) in offspring. Only a few studies have been conducted to date and results are conflicting. The current study investigates the associations between specific groups of prenatal maternal infections and offspring ADHD, considering timing of exposure and the role of fever.

**Methods:**

We used data from the prospective Norwegian Mother, Father and Child Cohort Study (MoBa), including more than 112,000 pregnancies, linked with data from the Medical Birth Registry of Norway and the Norwegian Patient Registry to estimate odds ratios for the likelihood that children develop ADHD after being exposed to maternal infections during gestation.

**Results:**

Children exposed to any maternal infection during pregnancy showed increased risk of receiving an ADHD diagnosis (OR = 1.15, CI = 1.03–1.27). Specifically, increased ADHD risk was observed after exposure to genitourinary infections in second (OR = 1.42, CI = 1.06–1.90) or third trimester (OR = 2.04, CI = 1.19–3.49), and to respiratory infections in second trimester (OR = 1.31, CI = 1.12–1.54), provided these infections were accompanied by episodes of fever. Increased ADHD risk was also observed after exposure to diarrhea without fever in the third trimester (OR = 1.25, CI = 1.07–1.46).

**Conclusions:**

Overall, our results suggest that prenatal exposure to maternal infections, particularly with co‐occurring episodes of fever, are risk factors for ADHD. Fever (or severity of the infection) appears to be more important in mid‐pregnancy associations. Our results indicate that type of infection and timing of exposure might influence the associations, but small effect sizes require careful interpretations. The association between infection and ADHD should be estimated using discordant siblings or other negative control designs that give better adjustment for unmeasured familial confounding.

## Graphical abstract

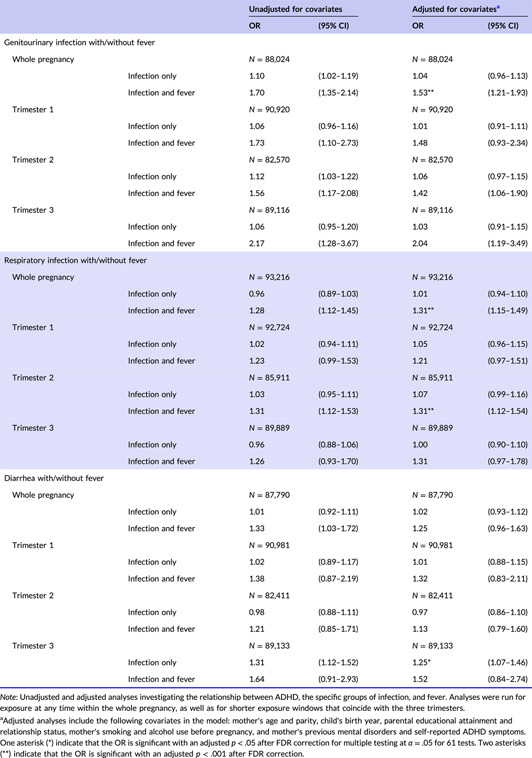

The current study investigates the associations between specific groups of prenatal maternal infections and offspring ADHD, taking into account timing of exposure and the role of fever. Results suggest that prenatal exposure to maternal infections, particularly with co‐occurring episodes of fever, are risk factors for ADHD but that type of infection and timing of exposure are important factors. Fever (or severity of the infection) appears to be more important in mid‐pregnancy associations. In late pregnancy diarrhea without co‐occurring fever was associated with increased risk of ADHD, suggesting there may be other aspects of such infections that contribute to pathogenesis nearer to term.

**Key points**

- Prenatal exposure to maternal infections, particularly with co‐occurring episodes of fever, are risk factors for ADHD.
- Type of infection and timing of exposure may be important factors.
- In mid‐pregnancy associations fever (or severity of the infection) appears to be important.
- In late pregnancy there may be other aspects of infections that contribute to pathogenesis.

## INTRODUCTION

Attention‐deficit hyperactivity disorder (ADHD), characterized by attention problems, hyperactivity, and impulsive behavior (American Psychiatric Association, 2013), is a prevalent neuropsychiatric condition in childhood, with a worldwide prevalence estimate of approximately 7.2% (Thomas et al., 2015).

Although ADHD is highly heritable, several environmental factors are associated with increased risk for a diagnosis (Thapar & Cooper, 2016). Neural development may be particularly vulnerable to environmental factors during the gestational period (Kim et al., 2015). Gestational risk factors include maternal infections and fever. Increasing evidence suggest that viral, bacterial and parasitic infections can trigger immune responses that increase the risk for subsequent neuropsychiatric disorders including autism (Jiang et al., 2016) and schizophrenia (Khandaker et al., 2012).

The risk of ADHD after in utero exposure to maternal infections has been studied less extensively, and findings are inconsistent. Some studies suggest that maternal infections in pregnancy are associated with ADHD in offspring (Lydholm et al., 2019; Mann & McDermott, 2011; Parker et al., 2016; Werenberg Dreier et al., 2016). Other studies have either failed to reveal an association between prenatal infection and ADHD (Chudal et al., 2019) or discovered that associations found at a population level were attenuated when adding sibling comparisons to the design (Ginsberg et al., 2019). Some report associations only for specific types of infections, like genitourinary (Mann & McDermott, 2011; Silva et al., 2014) or respiratory (Parker et al., 2016; Pineda et al., 2007). Several of the studies were hampered by limitations such as retrospective designs and insufficient sample size which may partly explain the inconsistent results.

The aversive effects due to prenatal exposures may be related to sensitive phases in gestational neurodevelopment (Kim et al., 2015). Thus far, studies have indicated sensitive phases in all three trimesters (Gustavson, Røysamb, & Borren, 2019; Lydholm et al., 2019; Werenberg Dreier et al., 2016). Differences in timing may explain conflicting results in previous studies.

The largest published study on ADHD and maternal infections and fever during pregnancy employed a prospective cohort of more than 89,000 pregnancies in Denmark (Werenberg Dreier et al., 2016). The overall associations between specific groups of infections and ADHD risk were small or non‐existent. However, when the exposures were considered during specific gestational periods, increased rates of ADHD were observed following fever in gestational Weeks 9–12, and genitourinary infections in Weeks 33–36. Interestingly, a previous study on the present sample by Gustavson, Ask, et al. (2019) has confirmed more cases of ADHD in children who were prenatally exposed to maternal fever in early pregnancy than in unexposed children. Gustavson's study did not take into account the specific types of infections though.

Strengths in the Danish study included large sample size allowing for detection of small associations in subcategories of the data (types of infections, timing of exposure), and prospective data collection mitigating against the potential for subjective bias. However, the study also had some limitations. For example, only 13% of the sample provided information on gestational Weeks 33–36. Moreover, as genetic transmission is central in the etiology of ADHD (Thapar & Cooper, 2016), an important limitation is that they did not adjust for parental symptoms or diagnosis of ADHD. With multiple testing some findings may also be coincidental.

In this study, we aim to replicate and extend the findings of Werenberg Dreier et al. (2016), using a large sample from the Norwegian Mother, Father and Child Cohort Study (MoBa) including more than 112,000 pregnancies. Our specific aims are to:

1. Estimate the association between prenatal exposure to any maternal infection during pregnancy and offspring risk of ADHD.
2. Estimate associations between groups of infections during pregnancy (genitourinary, respiratory, persistent viral infections and diarrhea), and offspring risk of ADHD.
3. Explore sensitive periods during gestation.
4. Investigate to what extent associations between maternal infections and ADHD risk differ according to the presence or absence of maternal fever.

## MATERIALS AND METHODS

### Participants

MoBa is a prospective pregnancy cohort study conducted by the Norwegian Institute of Public Health (Magnus et al., 2016). Participants were recruited from all over Norway from 1999 to 2008 (participation rate 41%). The cohort includes 114.500 children, 95.200 mothers and 75.200 fathers. This study is based on version 10 of the quality‐assured data files released for research in 2017. The establishment of MoBa and initial data collection was based on a license from the Norwegian Data Protection Agency and approval from The Regional Committees for Medical and Health Research Ethics and is now based on regulations related to the Norwegian Health Registry Act. The current study was approved by The Regional Committees for Medical and Health Research Ethics (2014/2266).

The information used is from maternal questionnaires completed at around gestational Week 17, gestational Week 30, and 6 months after birth.

The Medical Birth Registry (MBRN) is a national health registry containing information about all births in Norway. The MBRN provided information about year of birth, parity, as well as maternal age.

**FIGURE 1:**
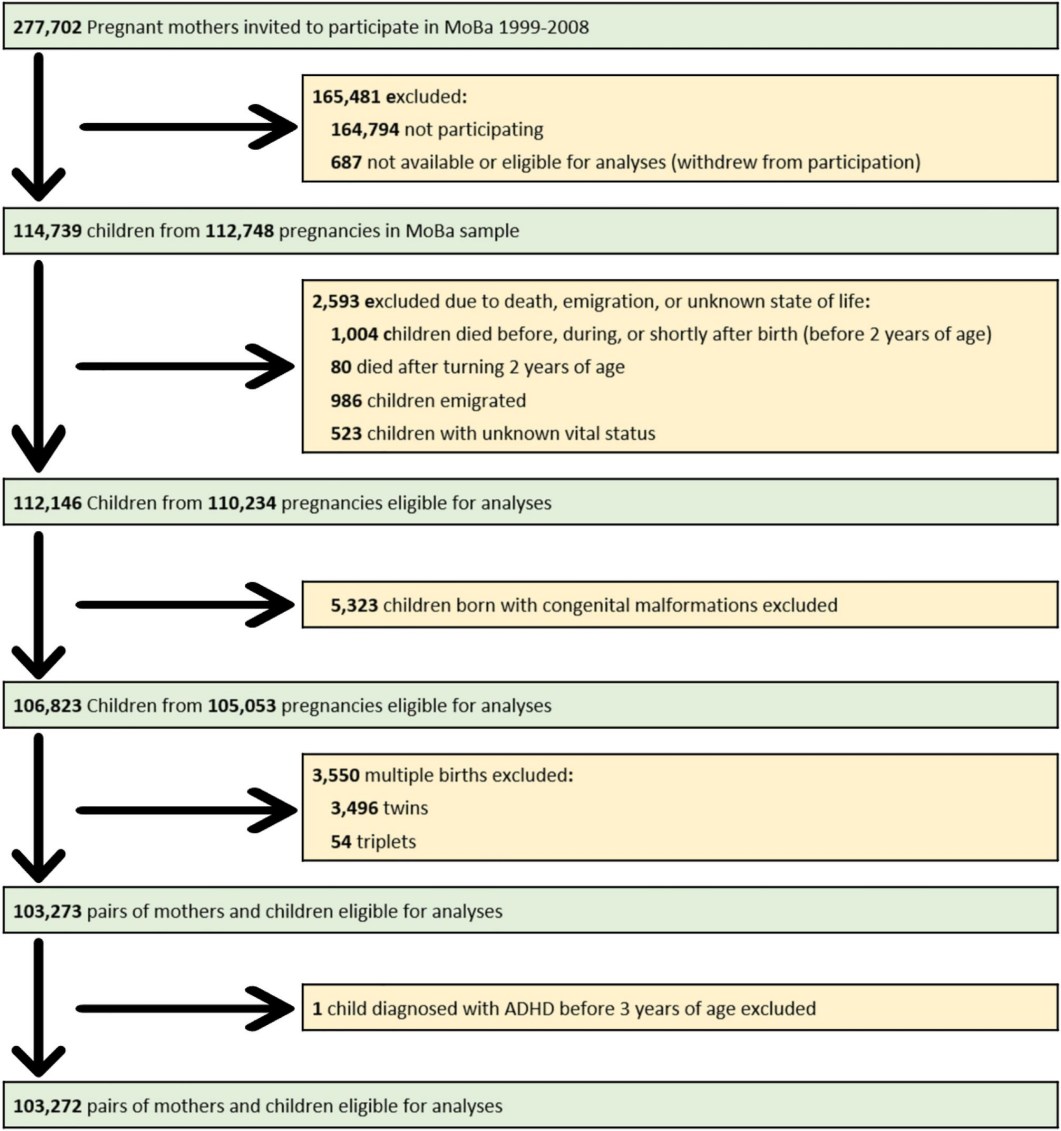
Flow diagram illustrating inclusion of participating mothers and children. ADHD indicates attention‐deficit hyperactivity disorder; MoBa, The Norwegian Mother, Father and Child Cohort Study

Twins are more likely to have low birth weight, which is an important determinant of neonatal outcome (Doom et al., 2012). Children from multiple births (*n* = 3.550) were excluded from the sample (Gustavson, Røysamb, & Borren, 2019). We also excluded children who died or emigrated (*n* = 2.593). A flow‐chart of participating mothers and children is presented in Figure 1.

### Measures

#### Outcome

Information about ADHD diagnoses was provided by data from the Norwegian Patient Registry (NPR). All government‐funded clinics in Norway report diagnoses according to the 10th revision of the International Classification of Diseases (ICD‐10) to the NPR, and since 2008 this information has been linkable to other data sources (including personal id numbers). Children registered at least once between the years 2008 and 2017 with Hyperkinetic disorder (F90) were classified as having ADHD. As the diagnostic uncertainty is high in the youngest age group, children with an ADHD diagnoses received before the age of 3 were excluded (*n* = 1).

#### Exposures

In each of the three questionnaires mothers indicated on a list of several infections which ones they had suffered from during pregnancy, mainly within 4‐week time windows (see Table S1 in Supporting Information S1 for details). At 17 weeks they reported for Weeks 0–12, at Week 30 they reported for Weeks 13–29, and 6 months after birth they reported for the remaining part of pregnancy, gestational weeks 30 until birth. Exceptions to this were urinary tract infections, which were only reported once between 0 and 12 gestational weeks, though in a similar fashion throughout the rest of the pregnancy, and pyelonephritis, which was reported only once for a larger time window of 0–12 gestational weeks. Infections were grouped in line with the classification of infections done in Werenberg Dreier et al. (2016) and Parker et al. (2016): (a) Any infection, including all the infections; (b) Genitourinary infections, including urinary tract infection (cystitis), pyelonephritis, and vaginal symptoms; (c) Persistent viral infections, including orofacial herpes infection, genital herpes infection and condylomas; (d) Respiratory infections, including influenza, pneumonia/bronchitis, ear infection, sinusitis, throat infection, common cold and other cough (other cough was only reported in 2 questionnaire); and (e) Diarrhea. Exposures were further defined by timing: the whole duration of pregnancy (from gestational Week 0 to birth), by trimester (trimester 1 = gestational Weeks 0–12; trimester 2 = 13–28; trimester 3 = 29 to birth), and during specific intervals of gestational time (gestational Weeks: 0–4, 5–8, 9–12, 13–16, 17–20, 21–24, 25–28, and 29 to the end of pregnancy). The definition of trimesters was the best fit with the reporting format of the questionnaires, deviating slightly from standard definitions.

Incidences of fever were also (except for the third trimester) reported within 4‐week time windows. In the first trimester, fever reaching a temperature of at least 38.5° was reported. In the second questionnaire, fever was registered with the highest temperature measured, and we used a cut‐off of 38.5° or more to define fever. For the third trimester, fever was reported with no specific information regarding temperature.

We created a separate set of exposure variables combining infections and fever on a nominal scale: (a) Infection only; (b) Infection and fever; (c) Neither fever nor infection. Due to sample size limitations, this combined exposure was not used when investigating the 4‐week intervals.

#### Covariates

Covariates were chosen based on results from previous studies investigating prenatal risk factors for ADHD (Ask et al., 2018; Gustavson, Røysamb, & Borren, 2019; Werenberg Dreier et al., 2016), provided that the variables were associated with both the exposure and outcome variables in our data, and that they were not thought to be potential mediators of the exposure‐outcome effect. The following covariates were included: mother's age and parity, child's birth year, parental educational attainment and relationship status, mother's smoking and alcohol use before pregnancy, and mother's previous mental disorders and self‐reported ADHD symptoms (measured by the 6‐item World Health Organization adult ADHD self‐report scale screener; Kessler et al., 2005). See supporting information for detailed information about covariates (Table S2 in Supporting Information S1) and handling of missing data (Supporting Information S1).

### Statistical analyses

Unadjusted and adjusted logistic regression models were run to estimate the offspring's odds of receiving an ADHD diagnosis after exposure to a maternal infection at any time during pregnancy and within specific gestational time windows, relative to unexposed children. Aiming to replicate and extend the findings of Werenberg Dreier et al. (2016), analyses were run for the overall infection variable and separately for the four infection groups (genitourinary infections, respiratory infections, diarrhea, and persistent viral infections), which were defined in line with the classification of infections in Werenberg Dreier et al. (2016) and Parker et al. (2016).

To investigate how the presence or absence of maternal fever play a role for the association between infections and ADHD, a separate set of logistic regression analyses were run, using exposure variables combining information on the four specific infection groups with fever exposure during the same time window. Odds ratios were calculated for the risk of receiving an ADHD diagnosis with and without exposure to infections with or without fever.

To correct for multiple testing, we applied a false discovery rate (FDR) correction at *α* = .05 for a total of 61 tests. This is quite a strict correction, as several of the exposure‐outcome associations tested partly overlap in the different models. Therefore, FDR correction was mainly applied to highlight robust findings and encourage caution in interpretation of weaker associations.

Statistical analyses were performed using IBM Statistical Package for Social Science Version 25 software (SPSS, Inc).

## RESULTS

### Descriptives

In our sample, 75,511 children had been prenatally exposed to any infection. By separating the four different infection groups, 35,271 children were prenatally exposed to genitourinary infections, 57,251 to respiratory infections, 20,759 to diarrhea, and 7,635 to persistent viral infections. Table S3 in Supporting Information S1 presents the rates of ADHD diagnosis in offspring exposed to the different infection groups. Table S4 in Supporting Information S1 presents the proportion of mothers with and without fever in the four infection groups, and the rates of ADHD diagnosis in their offspring.

### Main models

The results of the unadjusted and adjusted logistic regression models estimating overall associations between prenatal infections and ADHD are displayed in Table 1. After adjusting for covariates, an association between any infection and ADHD diagnoses (OR = 1.15, CI = 1.03–1.27, pFDR = 0.087) was found. When separating the four infection groups, an association with ADHD risk was observed only for respiratory infections (OR = 1.10, CI = 1.01–1.19, pFDR = 0.128).

**TABLE 1:** Odds ratios of attention‐deficit hyperactivity disorder (ADHD) and exposure to maternal infections during gestation

Group of infections (number of children prenatally exposed)	Exposed any time during pregnancy			
	OR unadjusted	(95% CI)	OR adjusted^a^	(95% CI)
Any infection (74,998)	1.15	(1.04–1.28)	1.15	(1.03–1.27)
Genitourinary infections (35,038)	1.18	(1.09–1.27)	1.07	(0.99–1.16)
Respiratory infections (56,841)	1.07	(0.98–1.15)	1.10	(1.01–1.19)
Diarrhea (20,604)	1.07	(0.98–1.17)	1.04	(0.95–1.14)
Persistent viral infections (7,580)	1.09	(0.96–1.24)	1.12	(0.99–1.26)

*Note*: Unadjusted and adjusted logistic regressions investigating the associations between gestational exposure to maternal infections and odds of receiving an ADHD diagnosis in childhood.

^a^ Adjusted analyses include the following covariates in the model: mother's age and parity, child's birth year, parental educational attainment and relationship status, mother's smoking and alcohol use before pregnancy, and mother's previous mental disorders and self‐reported ADHD symptoms.

Associations between infections during specific time windows and ADHD risk are shown in Table S5 in Supporting Information S1. Adjusted analyses of exposure to any infection, revealed an association after exposure in gestational Weeks 5–8 (OR = 1.12, CI = 1.02–1.22, pFDR = 0.099). Associations were also found after exposure in Weeks 21–24 (OR = 1.08, CI = 1.00–1.18, pFDR = 0.214), 25–28 (OR = 1.09, CI = 1.01–1.19, pFDR = 0.150), and 29‐birth (OR = 1.08, CI = 1.00–1.17, pFDR = 0.828). None of these associations remained significant after FDR‐corrections.

For the specific groups of infections, adjusted analyses showed increased odds for ADHD diagnoses in offspring after exposure to maternal genitourinary infections in gestational Weeks 5–8 (OR = 1.14, CI = 1.01–1.29, pFDR = 0.183), respiratory infections in gestational Weeks 13–16 (OR = 1.09, CI = 1.01–1. 19, pFDR = 0.183) and Weeks 21–24 (OR = 1.11, CI = 1.00–1.24, pFDR = 0.214), and diarrhea from gestational Week 29‐birth (OR = 1.25, CI = 1.08–1.45, pFDR <0.046), with only the last one significant after FDR corrections.

The results of the analyses including the fever‐infection combined exposure variable are presented in Table 2. The infection groups showed different patterns with regards to timing and the relevance of co‐occurring fever.

**TABLE 2:** Odds ratios of attention‐deficit hyperactivity disorder (ADHD) and exposure to infections with/without fever in specific time windows of the pregnancy

		Unadjusted for covariates		Adjusted for covariates^a^	
		OR	(95% CI)	OR	(95% CI)
Genitourinary infection with/without fever					
Whole pregnancy		*N* = 88,024		*N* = 88,024	
	Infection only	1.10	(1.02–1.19)	1.04	(0.96–1.13)
	Infection and fever	1.70	(1.35–2.14)	1.53**	(1.21–1.93)
Trimester 1		*N* = 90,920		*N* = 90,920	
	Infection only	1.06	(0.96–1.16)	1.01	(0.91–1.11)
	Infection and fever	1.73	(1.10–2.73)	1.48	(0.93–2.34)
Trimester 2		*N* = 82,570		*N* = 82,570	
	Infection only	1.12	(1.03–1.22)	1.06	(0.97–1.15)
	Infection and fever	1.56	(1.17–2.08)	1.42	(1.06–1.90)
Trimester 3		*N* = 89,116		*N* = 89,116	
	Infection only	1.06	(0.95–1.20)	1.03	(0.91–1.15)
	Infection and fever	2.17	(1.28–3.67)	2.04	(1.19–3.49)
Respiratory infection with/without fever					
Whole pregnancy		*N* = 93,216		*N* = 93,216	
	Infection only	0.96	(0.89–1.03)	1.01	(0.94–1.10)
	Infection and fever	1.28	(1.12–1.45)	1.31**	(1.15–1.49)
Trimester 1		*N* = 92,724		*N* = 92,724	
	Infection only	1.02	(0.94–1.11)	1.05	(0.96–1.15)
	Infection and fever	1.23	(0.99–1.53)	1.21	(0.97–1.51)
Trimester 2		*N* = 85,911		*N* = 85,911	
	Infection only	1.03	(0.95–1.11)	1.07	(0.99–1.16)
	Infection and fever	1.31	(1.12–1.53)	1.31**	(1.12–1.54)
Trimester 3		*N* = 89,889		*N* = 89,889	
	Infection only	0.96	(0.88–1.06)	1.00	(0.90–1.10)
	Infection and fever	1.26	(0.93–1.70)	1.31	(0.97–1.78)
Diarrhea with/without fever					
Whole pregnancy		*N* = 87,790		*N* = 87,790	
	Infection only	1.01	(0.92–1.11)	1.02	(0.93–1.12)
	Infection and fever	1.33	(1.03–1.72)	1.25	(0.96–1.63)
Trimester 1		*N* = 90,981		*N* = 90,981	
	Infection only	1.02	(0.89–1.17)	1.01	(0.88–1.15)
	Infection and fever	1.38	(0.87–2.19)	1.32	(0.83–2.11)
Trimester 2		*N* = 82,411		*N* = 82,411	
	Infection only	0.98	(0.88–1.11)	0.97	(0.86–1.10)
	Infection and fever	1.21	(0.85–1.71)	1.13	(0.79–1.60)
Trimester 3		*N* = 89,133		*N* = 89,133	
	Infection only	1.31	(1.12–1.52)	1.25*	(1.07–1.46)
	Infection and fever	1.64	(0.91–2.93)	1.52	(0.84–2.74)

*Note*: Unadjusted and adjusted analyses investigating the relationship between ADHD, the specific groups of infection, and fever. Analyses were run for exposure at any time within the whole pregnancy, as well as for shorter exposure windows that coincide with the three trimesters.

^a^ Adjusted analyses include the following covariates in the model: mother's age and parity, child's birth year, parental educational attainment and relationship status, mother's smoking and alcohol use before pregnancy, and mother's previous mental disorders and self‐reported ADHD symptoms. One asterisk (*) indicate that the OR is significant with an adjusted *p* < .05 after FDR correction for multiple testing at *α* = .05 for 61 tests. Two asterisks (**) indicate that the OR is significant with an adjusted *p* < .001 after FDR correction.

A robust association was observed between genitourinary infections *with fever* at any time during pregnancy and offspring ADHD (OR = 1.53, CI = 1.21–1.93, pFDR < −0.000). Similarly, ADHD odds were increased after genitourinary infections with fever during the second (OR = 1.42, CI = 1.06–1.90, pFDR = 0.128) and third trimester (OR = 2.04, CI = 1.19–3.49, pFDR = 0.087), though these were not significant after FDR‐correction. There was no association between exposure to maternal genitourinary infections without fever and offspring ADHD.

Children's odds for receiving an ADHD diagnosis were increased after prenatal exposure (at any time) to respiratory infections in combination with fever (OR = 1.31, CI = 1.15–1.49, pFDR < 0.000). This association appears to be driven by exposures during the second trimester (OR = 1.31, CI = 1.12–1.54, pFDR < 0.000).

Interestingly, while diarrhea at any time in pregnancy revealed no significant associations (neither with nor without fever), a closer look at the specific trimesters revealed a robust association for exposure to diarrhea alone during the third trimester (OR = 1.25, 95% CI = 1.07–1.46, pFDR < 0.049). Exposure to diarrhea with fever in the same trimester revealed no significant association.

No significant associations were observed between persistent viral infections and ADHD risk.

## DISCUSSION

In this study, prenatal exposure to maternal infections during pregnancy was associated with increased risk of ADHD. Overall, patterns were similar both with regards to estimates for different time points and types of infections. Effect sizes were in general small in the present study, but comparable to findings in other prospective cohorts (Lydholm et al., 2019; Mann & McDermott, 2011; Werenberg Dreier et al., 2016). There is a possibility that type of infection as well as the timing of exposure may be important factors. The risk of ADHD was increased when infection was accompanied by fever, for second trimester infections, particularly for genitourinary infections and respiratory infections. Genitourinary infections with fever in the third trimester were also associated with increased risk of ADHD in offspring. Third trimester exposure to maternal diarrhea did not need co‐occurrence of fever to show an association with offspring ADHD. Small effect sizes and generally similar patterns imply careful interpretations though.

Exposure to any infection during pregnancy was associated with a small increase in risk of ADHD diagnoses in offspring in our study. Werenberg Dreier et al. (2016) did not find similar results. However, their findings suggest that both gestational timing and the specific type of infection may be of relevance. Our results also reveal associations for specific groups of infections and ADHD, and points to phases of vulnerability for maternal infection exposure both in early‐ (Weeks 5–8), mid‐ (Weeks 13–16), and late‐ pregnancy (Weeks 21–24 and 29‐birth), with timing depending on the type of infection. It should be noted though, that patterns of estimates are quite similar across different time windows and types of infection, although they are not all statistically significant.

The importance of certain infection groups and vulnerable phases during gestation have been suggested previously (Silva et al., 2014; Werenberg Dreier et al., 2016). Werenberg Dreier et al. (2016) found a heightened risk (Hazard ratio of 1.60) of ADHD in offspring after exposure to maternal genitourinary infections during late gestation (Weeks 33–36) after controlling for episodes of fever. Although we also observed associations in both second and third trimesters between genitourinary infections and ADHD, these associations were limited to genitourinary infections combined with fever and not observed for genitourinary infections alone. Other studies reporting an association between genitourinary infections and ADHD (Mann & McDermott, 2011; Silva et al., 2014) do not control for effects of fever, and do not investigate the role of exposure timing.

Our overall analyses of maternal respiratory infections suggest that the odds for ADHD in offspring were increased by 31% after exposure any time during gestation if accompanied by fever. Previous studies on maternal respiratory infections and ADHD risk have been inconclusive, with some studies supporting an association (Parker et al., 2016; Pineda et al., 2007), and Werenberg Dreier et al. (2016) reporting no such association. Our results indicate gestational Weeks 13–16 to be a vulnerable time for the fetus to be exposed to maternal respiratory infections, as well as gestational Weeks 21–24. However, taking fever into account, we found no associations for respiratory infections without fever in any trimester, which is in line with Werenberg Dreier et al. (2016), as they controlled for fever. Also, since respiratory infections with fever was associated with higher odds of ADHD, we cannot rule out that the severity of the respiratory infections condition (as reflected by febrile episodes) may play a role in this association. The pattern of our results could also indicate that the fever rather than the respiratory infection per se, represent the relevant mechanisms behind the associations.

Neither Werenberg Dreier et al. (2016) nor our study found significant associations between gestational diarrhea exposure overall and ADHD. However, considering the timing of exposure, our study found that children whose mothers had diarrhea in third trimester showed higher rates of ADHD diagnoses later in life compared to unexposed children. This association did not depend on co‐occurring episodes of fever. Werenberg Dreier et al. (2016) did not report any time‐specific analyses for diarrhea.

Diarrhea may occur with many types of viral, bacterial, or parasitic infections and few studies have investigated adverse outcomes related to the specific symptom diarrhea. Prenatal exposure to maternal infections with diarrhea have been linked with increased risk for the baby to be small for gestational age (Newman et al., 2019), a suggested risk factor for developing ADHD (Heinonen et al., 2010). Being born small for gestational age may therefore be a mediating factor for the association observed in our study.

In line with Werenberg Dreier et al. (2016), we did not observe associations between persistent viral infections and ADHD risk. Some studies have indicated that other types of maternal viral infections during pregnancy may be associated with ADHD, such as Arpino et al. (2005) who found an unexpected high rate of viral rash from exanthematic diseases during pregnancy in mothers of children with ADHD. Also, severe respiratory viral infections (flu) during pregnancy were considered a risk factor for offspring ADHD by Pineda et al. (2007).

Our results point to vulnerable phases particularly in second and third trimesters. The second trimester was associated with vulnerability to genitourinary or respiratory infections combined with fever, while the third trimester was associated with vulnerability to genitourinary infections with fever and to diarrhea alone.

Respiratory infections with fever represent a risk factor in the second trimester. First and second trimester specific effects have been suggested in previous MoBa studies. Gustavson, Ask, et al. (2019) found risk for offspring ADHD associated with first‐ and second‐trimester fever, while Hornig et al. (2018) linked maternal fever in the second trimester with risk of autism spectrum disorders in offspring. In the middle of the second trimester neurons begin to migrate to their final locations in cortex, involving particularly active processes of formation and connections of dendrites, which signifies the start of a period of massive brain development (Dombrowski et al., 2003). Environmental disturbances may influence the timing of these processes and lead to subtle malformations of the central nervous system (CNS), later expressed through psychological outcomes (Dombrowski et al., 2003; Shi et al., 2003). Strickland (2014) proposes that particularly the last part of pregnancy (gestational Weeks 32–40) may constitute a period of heightened vulnerability towards insults from maternal infections or inflammatory responses with regards to offspring risk of ADHD. We found that maternal diarrhea in the third trimester was associated with increased ADHD risk. Lammertink et al. (2021) have also described the third trimester as a vulnerable phase due to fast‐developing cellular processes taking place (synaptogenesis, neuronal migration, and myelination), regulating neural circuits. Environmental disturbances may contribute to altered neural programming, affecting crucial systems like the hypothalamic—pituitary—adrenal axis and the autonomic nervous system, important for the etiology of several psychopathologies (Lammertink et al., 2021).

Associations between maternal infections and neurodevelopmental outcomes in offspring may be explained by the specific infective agents themselves, or mechanisms of the maternal immune activation that typically follows, such as the elevated temperature levels of fever (hyperthermia). Fever could also be an indicator of elevated levels of pro‐inflammatory cytokines and other molecules (Gustavson, Røysamb, & Borren, 2019; Romanovsky et al., 2005), which may cross the placenta and influence fetal neurodevelopment through other mechanisms than hyperthermia (Flinkkilä et al., 2016; Gustavson, Røysamb, & Borren, 2019).

This study found that both genitourinary infections and respiratory infections were associated with offspring ADHD only when episodes of fever co‐occurred with the infection, suggesting either, that the association with ADHD is driven by more severe cases of genitourinary infections and respiratory infections, or by fever itself. It has been suggested that fever may contribute in the assumed disruption of a healthy CNS development (Edwards, 2006; Flinkkilä et al., 2016; Gustavson, Røysamb, & Borren, 2019). Antipyretic medication (e.g., acetaminophen), has also been suggested to play a role. However, previous studies report mixed findings with regards to the involvement of acetaminophen (Gustavson, Røysamb, & Borren, 2019; Werenberg Dreier et al., 2016; Ystrom et al., 2017).

Werenberg Dreier et al. (2016) found that exposure to fever early in pregnancy (Weeks 9–12) was associated with a higher rate of ADHD in offspring. Previous studies on the present sample also suggest that maternal fever in early and mid‐pregnancy may be a risk factor for neurodevelopmental disorders (Gustavson, Røysamb, & Borren, 2019; Hornig et al., 2018). In this study, diarrhea was the only group of infections associated with ADHD in offspring without depending on co‐occurring fever. Diarrhea was also one of two groups of infections (diarrhea and genitourinary infections) associated with offspring ADHD after occurrence in the latter part of pregnancy. Our findings may therefore suggest that fever and/or associated processes may play an important role in mid‐pregnancy, while other mechanisms linked with diarrhea or genitourinary infections may explain the vulnerability of neurodevelopment in the third trimester. This would also be in line with Werenberg Dreier et al. (2016) who reported increased risk of ADHD in offspring after exposure to maternal genitourinary infections late in pregnancy. It may appear strange then that genitourinary infections in the third trimester did require co‐occurring fever to reveal associations with ADHD while fever was not required in the diarrhea association. An explanation could be that for the more severe cases of genitourinary infections, another aspect than fever itself, one that correlates with fever, could be relevant in explaining the association between third trimester genitourinary infections and ADHD. This interpretation fits with the results of Werenberg Dreier et al. (2016) as they report an association independent of fever in the last part of pregnancy.

Overall, it should be pointed out, that the different results in the present study appear to follow a similar pattern and the effect sizes are in general small. Some differences between statistical significance estimates could be due to differences in sample size. However, effect sizes presented here are in proportion with those reported in several other prospective cohort studies investigating associations between in utero exposures and child outcomes (Lydholm et al., 2019; Mann & McDermott, 2011; Werenberg Dreier et al., 2016), and studies conducted prospectively have often been found to give weaker estimates than those with retrospective assessments (Dreier et al., 2014). It is still early to determine with certainty whether effects are in fact infection‐specific and whether they are timing‐dependent, but the current results provide some indications that should be investigated further.

This study has several strengths. We used data from a very large birth cohort, allowing us to reveal small differences in ADHD rates between groups and power to investigate exposure timing and fever in detail. The data were prospectively collected and included several pregnancy exposures, not normally available in the patient registers. With multiple testing there is a risk that some of the reported findings could be due to chance. Therefore, to highlight the more robust findings, and to emphasize caution in the interpretation of weaker associations, we applied FDR corrections to the alpha levels.

Our results should be interpreted considering some important limitations. First, MoBa has a participation rate of 41%, and low participation rates may lead to selection bias. Studies have found that women who participate in cohort studies (MoBa included) are usually healthier, better educated, etc., than the underlying source population (Nilsen et al., 2009, 2013). However, the same studies found no statistically significant differences in measures of studied exposure‐outcome associations between participants in MoBa and the total population, suggesting for reasonable generalizability. Additionally, data simulation studies suggest that estimates of associations are more robust against under‐representation of some groups, than are estimates of means and frequencies (Gustavson, Røysamb, & Borren, 2019; Gustavson et al., 2012). Second, analyses for the whole pregnancy includes all mothers who reported to have had an infection in any of the three questionnaires, regardless of whether they failed to respond to all three questionnaires. This is a potential limitation, as false negatives could have washed out some of the true effect. Third, the oldest children of the sample were 8 years old the year that the NPR started recording individual identifiable diagnoses. Accordingly, some of these children may have been diagnosed with ADHD before that. If they were not again in contact with specialist health care between the years 2008 and 2017, they may appear as false negatives in the present study. Fourth, it has been suggested that infants who are later in life diagnosed with ADHD could have more regulatory problems (e.g., excessive crying, difficulties sleeping and feeding) placing a greater demand on parents beginning in infancy (Hemmi et al., 2011). We cannot rule out the possibility of recall bias for third trimester associations, as third trimester exposures were reported on the postnatal 6‐month questionnaire. However, third trimester reports were not required to be as specific with regards to timing since these infections were reported for the whole last trimester, while infection reports of the two other trimesters were reported more specifically for 4 weeks time‐windows. Fifth, there is a chance that a woman may have had several types of infections during the same time window, and if a fever is registered within the same time window, it would be difficult to tell with which infection came the fever. Sixth, although we adjusted the analyses for several measured covariates, unmeasured confounding may be present. For example, unmeasured aspects of living conditions, social adversity, maternal personality, or family lifestyle that could be both genetically and environmentally influenced, might increase the likelihood of both maternal infections and ADHD risk in offspring. Confounding induced by such shared familial risk factors may be addressed in future studies by use of sibling discordance analyses (Donovan & Susser, 2011) or in molecular genetic studies (Pingault et al., 2018). Seventh, our exposure measures were based on maternal self‐report. Self‐reported infections and events of fever may have low reliability and thus attenuate association estimates. Eighth, systematic biases may also affect results if over‐ or under‐reporting of infections is related to maternal ADHD. ADHD is highly heritable and the estimated associations between maternal prenatal infections and ADHD may be falsely inflated or deflated if mothers with ADHD for some reason systematically over‐ or under‐report infections more frequently than mothers without ADHD. However, we adjusted the analyses for mothers' self‐reported ADHD symptoms to avoid this potential confounding. Ninth, the covariate maternal ADHD symptoms had a large proportion of missing values. Multiple imputations were performed, using predictors related to ADHD such as education, smoking and drinking habits, marital status, and so forth. However, the assumption in multiple imputations that missingness is random may be violated. For example, women with higher levels of ADHD symptoms may have failed to respond to the relevant questionnaire more often than women with lower levels over and above what was accounted for by the predictors in the imputation. This means that associations observed may potentially still be confounded by maternal ADHD. Finally, a challenge that in general concerns most studies on effects of infections, is the need to separate the effects of infections, fever, and their treatments from each other (Werenberg Dreier et al., 2016). Although our findings indicate timing‐specific associations, it must be emphasized that caution must be applied when interpreting these results, as they require replication.

## CONCLUSIONS

Our results suggest that prenatal exposure to maternal infections is associated with elevated risk of ADHD in the offspring. While there were clearly similarities between estimates for the different time points and types of infections, some infections and some timepoints revealed stronger associations. This could indicate that type of infection and gestational timing of exposure may be important factors, though the small effect sizes imply careful interpretation. Fever (or associated processes) could be a key factor in associations observed between genitourinary infections and respiratory infections in mid‐pregnancy and risk of ADHD, although such associations could also be driven by infection severity. In the late‐pregnancy associations between diarrhea/genitourinary infections and risk of ADHD it appears that febrile episodes may not be equally relevant, and that other aspects of the infections themselves may be key to the associations. Our results require replication in future population‐based prospective studies.

## CONFLICT OF INTERESTS

The authors have declared that they have no competing or potential conflicts of interest.

## ETHICAL CONSIDERATIONS

The current study was approved by The Regional Committees for Medical and Health Research Ethics (2014/2266).

## AUTHOR CONTRIBUTIONS

**Kjersti M. Walle:** Conceptualization; Data curation; Formal analysis; Investigation; Methodology; Project administration; Writing ‐ original draft; Writing ‐ review and editing. **Ragna B. Askeland:** Conceptualization; Funding acquisition; Investigation; Methodology; Project administration; Resources; Writing ‐ original draft; Writing ‐ review and editing. **Kristin Gustavson:** Conceptualization; Data curation; Formal analysis; Funding acquisition; Investigation; Methodology; Writing ‐ original draft; Writing ‐ review and editing. **Eivind Ystrom:** Conceptualization; Investigation; Methodology; Writing ‐ original draft; Writing ‐ review and editing. **Michaeline Bresnahan:** Conceptualization; Funding acquisition; Investigation; Methodology; Writing ‐ original draft; Writing ‐ review and editing. **Mady Hornig:** Conceptualization; Funding acquisition; Investigation; Methodology; Writing ‐ original draft; Writing ‐ review and editing. **Ezra S. Susser:** Conceptualization; Funding acquisition; Investigation; Methodology; Writing ‐ original draft; Writing ‐ review and editing. **Camilla Stoltenberg:** Conceptualization; Data curation; Funding acquisition; Investigation; Methodology; Project administration; Writing ‐ original draft; Writing ‐ review and editing. **Ted reichborn‐kjennerud:** Conceptualization; Data curation; Funding acquisition; Investigation; Methodology; Project administration; Supervision; Writing ‐ original draft; Writing ‐ review and editing. **Helga Ask:** Conceptualization; Formal analysis; Investigation; Methodology; Supervision; Validation; Writing ‐ original draft; Writing ‐ review and editing.

## Supporting information

Supporting Information S1

## Data Availability

The consent given by the participants does not open for storage of data on an individual level in repositories or journals. Researchers who want access to data sets for replication should submit an application to datatilgang@fhi.no. Access to data sets requires approval from The Regional Committee for Medical and Health Research Ethics in Norway and an agreement with MoBa.
