# Supplementary material for: Risk of attention‐deficit hyperactivity disorder in offspring of mothers with infections during pregnancy
Source: JCPP Adv. 2022 Mar 10;2(2):e12070. doi: 10.1002/jcv2.12070 (PMC10242954; doi:10.1002/jcv2.12070)
Supplement: Supplementary file 1 — Supporting Information S1 [file JCV2-2-e12070-s001.docx]

# **Supporting Information**

# **Risk of ADHD in offspring of mothers with infections during pregnancy**

Kjersti Mæhlum Walle, Ragna Bugge Askeland, Kristin Gustavson, Siri Mjaaland, Eivind Ystrom, W. Ian Lipkin, Per Magnus, Camilla Stoltenberg, Ezra Susser, Michaeline Bresnahan, Mady Hornig, Ted Reichborn-Kjennerud, Helga Ask.

**Table S1.** Types of infection and how they were reported in questionnaires for different time windows.

| **Infection group:** | **First trimester** | **Second trimester** | **Third trimester** |
| --- | --- | --- | --- |
|  |  |  |  |
| **Genitourinary infections:** |  |  |  |
| urinary tract infection | weeks 0-12 | weeks 13-16, 17-20, 21-24, 25-28, | weeks 29-birth |
| pyelonephritis | weeks 0-12 | - | - |
| vaginal catarrh | weeks 0-4, 5-8, 9-12 | weeks 13-16, 17-20, 21-24, 25-28, | vaginal catarrh & thrush reported together for weeks 29-birth |
| vaginal thrush | weeks 0-4, 5-8, 9-12 | weeks 13-16, 17-20, 21-24, 25-28, |  |
|  |  |  |  |
| **Persistent viral infections:** |  |  |  |
| orofacial herpes infection | weeks 0-12 | - | - |
| genital herpes infection | weeks 0-12 | - | - |
| Condylomas | weeks 0-12 | - | - |
|  |  |  |  |
| **Respiratory infections:** |  |  |  |
| influenza | weeks 0-4, 5-8, 9-12 | weeks 13-16, 17-20, 21-24, 25-28, | weeks 29-birth |
| pneumonia/bronchitis | weeks 0-4, 5-8, 9-12 | weeks 13-16, 17-20, 21-24, 25-28, | weeks 29-birth |
| ear infection / sinusitis | weeks 0-4, 5-8, 9-12 | weeks 13-16, 17-20, 21-24, 25-28, | sinusitis, ear & throat infection reported together for weeks 29-birth |
| throat infection | weeks 0-4, 5-8, 9-12 | weeks 13-16, 17-20, 21-24, 25-28, |  |
| common cold | weeks 0-4, 5-8, 9-12 | weeks 13-16, 17-20, 21-24, 25-28, | weeks 29-birth |
| other cough | - | weeks 13-16, 17-20, 21-24, 25-28, | - |
|  |  |  |  |
| **Diarrhea** | weeks 0-4, 5-8, 9-12 | weeks 13-16, 17-20, 21-24, 25-28, | weeks 29-birth |

**Table S2. Parental characteristics.**

|  | Mean (SD) or percentage | | n |
| --- | --- | --- | --- |
| Child’s birth year | | 2005 (2.215) | 103272 |
| Mother’s age | | 30.1 (4.667) | 103272 |
| Mother’s ADHD symptoms' score | | 2.1 (0.576) | 52632 |
| Missing data on mothers’ ADHD symptoms’ scores | | 49.04 % | 50640 |
| Highest level of education among parents:  Less than high school graduate | | 3.30 % | 88718 |
| High school graduate | | 22.30 % |  |
| Undergraduate education completed | | 32.70 % |  |
| Postgraduate education (masters or doctorate) completed | | 27.50 % |  |
| Missing | | 14.10 % | 14554 |
| Parents’ relationship status:  Married or in a relationship | | 87.10 % | 93035 |
| Single | | 3.00 % |  |
| Missing | | 9.90 % | 10237 |
| Parity:  First born | | 43.60 % | 103272 |
| Second born | | 36.20 % |  |
| Third (or more) born | | 20.20 % |  |
| Smoking before pregnancy  Yes | | 25.30 % | 91712 |
| No | | 63.50 % |  |
| Missing | | 11.20 % | 11560 |
| Alcohol use before pregnancy:  No | | 6.30 % | 86820 |
| less than 3 units per month | | 55.40 % |  |
| 1-3 units per week | | 21.50 % |  |
| 4-7 units per week | | 0.90 % |  |
| Missing | | 15.90 % | 16452 |
| Mother’s previous mental disorders  Previously diagnosed with eating disorders, depression, or anxiety | | 18.40 % | 103272 |
| No previous diagnosis | | 81.60 % |  |

**Appendix S1. Supplementary Methods: Covariates**

Table S2 presents descriptive statistics for all covariates included in the models, including percentage of missing data. The mothers’ ADHD symptoms were reported in a later questionnaire, filled out when the child was around 36 months of age. Due to participant dropout, information on ADHD symptoms were missing in 49.6% of the pregnancies. Assuming that these values were missing at random, we performed multiple imputation using the Markov chain Monte Carlo (MCMC) method, creating 5 data sets (Supplementary Methods in the online Supplement). All covariates were included in the imputation model in addition to the exposure and outcome variables.

For the other variables with lower levels of missing values (education, relationship status, smoking and alcohol use), missing values were included in a separate category of the nominal scales.

**Table S3.** Proportion of participating mothers by exposure to infections and ADHD rates in offspring.

|  | | ADHD | | | | Total |
| --- | --- | --- | --- | --- | --- | --- |
|  |  | No | | Yes | |  |
| Genitourinary infection | No | 46912 | 56.2 % | 1514 | 1.8 % | 48426 |
|  | Yes | 33753 | 40.4 % | 1285 | 1.5 % | 35038 |
| Total | | 80665 |  | 2799 |  | 83464 |
| Respiratory infection | No | 28352 | 32.9 % | 938 | 1.1 % | 29290 |
|  | Yes | 54907 | 63.7 % | 1934 | 2.2 % | 56841 |
| Total | | 83259 |  | 2872 |  | 86131 |
| Diarrhea | No | 58129 | 72.0 % | 1963 | 2.4 % | 60092 |
|  | Yes | 19886 | 24.6 % | 718 | 0.9 % | 20604 |
| Total | | 78015 |  | 2681 |  | 80696 |
| Persistent viral infection | No | 83014 | 88.8 % | 2890 | 3.1 % | 85904 |
|  | Yes | 7303 | 7.8 % | 277 | 0.3 % | 7580 |
| Total | | 90317 |  | 3167 |  | 93484 |
| Any infection | No | 14207 | 15.8 % | 439 | 0.5 % | 14646 |
|  | Yes | 72417 | 80.8 % | 2581 | 2.9 % | 74998 |
| Total | | 86624 |  | 3020 |  | 89644 |

Note: Number of cases prenatally exposed and unexposed to infections of the different groups and how many of these who were later diagnosed with ADHD.

**Table S4.** Proportion of participating mothers by exposure to infections and/or fever and ADHD rates in offspring.

| Genitourinary infections | | ADHD | | | | Total |
| --- | --- | --- | --- | --- | --- | --- |
|  |  | No | | Yes | |  |
| Whole pregnancy | No infection or fever | 55984 | 63.6 % | 1856 | 2.1 % | 57840 |
|  | Infection only | 27693 | 31.5 % | 1011 | 1.1 % | 28704 |
|  | Infection and fever | 1401 | 1.6 % | 79 | 0.1 % | 1480 |
|  | Total | 85078 | 96.7 % | 2946 | 3.3 % | 88024 |
| Respiratory infections | | ADHD | | | | Total |
|  |  | No | | Yes | |  |
| Whole pregnancy | No infection or fever | 38260 | 41.0 % | 1335 | 1.4 % | 39595 |
|  | Infection only | 45054 | 48.3 % | 1508 | 1.6 % | 46562 |
|  | Infection and fever | 6758 | 7.2 % | 301 | 0.3 % | 7059 |
|  | Total | 90072 | 96.6 % | 3144 | 3.4 % | 93216 |
| Diarrhea | | ADHD | | | | Total |
|  |  | No | | Yes | |  |
| Whole pregnancy | No infection or fever | 67111 | 76.4 % | 2300 | 2.6 % | 69411 |
|  | Infection only | 16385 | 18.7 % | 569 | 0.6 % | 16954 |
|  | Infection and fever | 1363 | 1.6 % | 62 | 0.1 % | 1425 |
|  | Total | 84859 | 96.7 % | 2931 | 3.3 % | 87790 |

Note: Number of cases prenatally unexposed or exposed to infection, infection and fever, or fever, and how many of these who were later diagnosed with ADHD.

**Table S5.** Exposure to infections in specific pregnancy time windows and risk of ADHD.

| **Infection group** | **OR unadjusted** | **(95% C.I.)** | **OR adjusted^1^** | **(95% C.I.)** |
| --- | --- | --- | --- | --- |
| **Any infection** |  |  |  |  |
| Weeks 0-4 | 1.03 | (0.92-1.15) | 1.00 | (0.90-1.12) |
| Weeks 5-8 | 1.12 | (1.02-1.22) | 1.12 | (1.02-1.22) |
| Weeks 9-12 | 1.01 | (0.93-1.10) | 1.01 | (0.93-1.10) |
| Weeks 13-16 | 1.07 | (1.00-1.15) | 1.07 | (0.99-1.15) |
| Weeks 17-20 | 1.02 | (0.93-1.12) | 1.03 | (0.93-1.13) |
| Weeks 21-24 | 1.07 | (0.98-1.16) | 1.08 | (1.00-1.18) |
| Weeks 25-28 | 1.09 | (1.01-1-18) | 1.09 | (1.01-1.19) |
| Weeks 29-birth | 1.09 | (1-00-1.18) | 1.08 | (1.00-1.17) |
| **Genitourinary infections** |  |  |  |  |
| Weeks 0-4 | 1.02 | (0.86-1.21) | 0.94 | (0.80-1.12) |
| Weeks 5-8 | 1.18 | (1.04-1.33) | 1.14 | (1.01-1.29) |
| Weeks 9-12 | 0.96 | (0.86-1.08) | 0.94 | (0.83-1.05) |
| Weeks 13-16 | 1.08 | (0.98-1.19) | 1.03 | (0.93-1.13) |
| Weeks 17-20 | 1.06 | (0.94-1.20) | 1.03 | (0.91-1.16) |
| Weeks 21-24 | 1.10 | (0.98-1.23) | 1.07 | (0.96-1.20) |
| Weeks 25-28 | 1.10 | (0.98-1.22) | 1.06 | (0.95-1.19) |
| Weeks 29-birth | 1.12 | (1.00-1.25) | 1.06 | (0.94-1.19) |
| **Respiratory infections** |  |  |  |  |
| Weeks 0-4 | 1.08 | (0.93-1.25) | 1.08 | (0.93-1.25) |
| Weeks 5-8 | 1.07 | (0.95-1.20) | 1.09 | (0.97-1.22) |
| Weeks 9-12 | 1.05 | (0.95-1.16) | 1.07 | (0.96-1.18) |
| Weeks 13-16 | 1.07 | (0.98-1.16) | 1.09 | (1.01-1.19) |
| Weeks 17-20 | 1.04 | (0.92-1.18) | 1.08 | (0.96-1.23) |
| Weeks 21-24 | 1.06 | (0.96-1.18) | 1.11 | (1.00-1.24) |
| Weeks 25-28 | 1.05 | (0.95-1.15) | 1.09 | (0.99-1.20) |
| Weeks 29-birth | 1.00 | (0.91-1.10) | 1.02 | (0.93-1.12) |
| **Diarrhea** |  |  |  |  |
| Weeks 0-4 | 1.18 | (0.91-1.52) | 1.13 | (0.87-1.46) |
| Weeks 5-8 | 1.09 | (0.90-1.32) | 1.08 | (0.89-1.31) |
| Weeks 9-12 | 1.03 | (0.87-1.23) | 1.01 | (0.85-1.21) |
| Weeks 13-16 | 1.10 | (0.95-1.26) | 1.07 | (0.93-1.23) |
| Weeks 17-20 | 0.79 | (0.60-1.03) | 0.79 | (0.60-1.03) |
| Weeks 21-24 | 0.99 | (0.80-1.22) | 0.97 | (0.78-1.19) |
| Weeks 25-28 | 1.16 | (0.98-1.37) | 1.09 | (0.92-1.29) |
| Weeks 29-birth | 1.33 | (1.15-1.54) | 1.25* | (1.08-1.45) |

Note: Unadjusted and adjusted timing specific odds ratios of the logistic regressions examining associations between gestational exposure to maternal infections and ADHD risk. ^1^ Adjusted for mother’s age and parity, child’s birth year, parental educational attainment and relationship status, mother's smoking and alcohol use before pregnancy, and the mother’s previous mental disorders and ADHD symptoms. One asterisk (*) indicate that the OR is significant with an adjusted p < .05 after FDR correction for multiple testing at α = .05 for 61 tests.
